# Constraints on crustal compositional architecture across the North China–Altaids transition and implications for craton margin reworking

**DOI:** 10.1093/nsr/nwae171

**Published:** 2024-05-15

**Authors:** Zhuo Ye, Xiaomiao Tan, Rui Gao, Qiusheng Li, Hongshuang Zhang, Xiaoyang Wu, Wenhui Li, Yingkang Li

**Affiliations:** SinoProbe Center, Chinese Academy of Geological Sciences, Beijing 100037, China; Key Laboratory of Deep-Earth Dynamics, Ministry of Natural Resources, Institute of Geology, Chinese Academy of Geological Sciences, Beijing 100037, China; School of Earth Sciences and Engineering, Sun Yat-sen University, Guangzhou 510275, China; School of Earth Sciences and Engineering, Sun Yat-sen University, Guangzhou 510275, China; Key Laboratory of Deep-Earth Dynamics, Ministry of Natural Resources, Institute of Geology, Chinese Academy of Geological Sciences, Beijing 100037, China; Key Laboratory of Deep-Earth Dynamics, Ministry of Natural Resources, Institute of Geology, Chinese Academy of Geological Sciences, Beijing 100037, China; Key Laboratory of Marine Geology and Environment, Institute of Oceanology, Chinese Academy of Sciences, Qingdao 266071, China; Key Laboratory of Deep-Earth Dynamics, Ministry of Natural Resources, Institute of Geology, Chinese Academy of Geological Sciences, Beijing 100037, China; China Geological Sample Information Center, China Geological Survey, Yanjiao 065201, China

**Keywords:** North China Craton, Altaids, crustal composition, receiver function, joint inversion, continental reworking

## Abstract

The phase of secular evolution of continents is manifested as the degree of compositional differentiation, modification and maturation of continental crusts, which is vital in understanding the mechanism of continental evolution but is difficult to quantify. Here we use integrated passive- and active-source seismic profiling to conduct joint analysis and inversion and derive Vs and Vp/Vs section models across the North China Craton (NCC) to southeastern Altaids boundary zone that bears a tectonic transition from a reworked ancient craton margin to a Phanerozoic accretionary orogen. We systematically exploited the imaged multiple physical properties as precise and delicate proxies to constrain the compositional architecture in the crust across this important tectonic transition subject to various crustal evolutional phases. Our Vs and Vp/Vs imaging, together with the existing isotopic data, characterizes the Yin Shan–Yan Shan belt as the northern NCC margin with layered homogeneous compositions that point to an evolved crust. However, the lower-crustal low-Vs/high-Vp/Vs signature that overlaps the shallowly dipping to horizontal reflective fabrics suggests that the crust of the northern NCC margin has undergone considerable reworking through lower-crustal-stretching-assisted melt migration and mixing since the late Paleozoic to Mesozoic eras. The process probably involved crust–mantle interaction and thus resulted in a compositionally modified ancient crustal basement. On the contrary, the southeastern Altaids domain manifests crustal complexity in compositions and structures inferred to be indicative of a juvenile crust of the Phanerozoic accretionary orogen. Our results provide deep physical-property constraints that shed new light on the crustal evolution of a complex craton margin.

## Teaser

The compositional differentiation and modification of continental crusts manifest the physical and chemical process reflecting the mechanism of secular evolution of continents; however, it is difficult to quantify. This work provides delicate seismic constraints to address this issue in the complex craton margin of North China.

## INTRODUCTION

**Figure 1.**
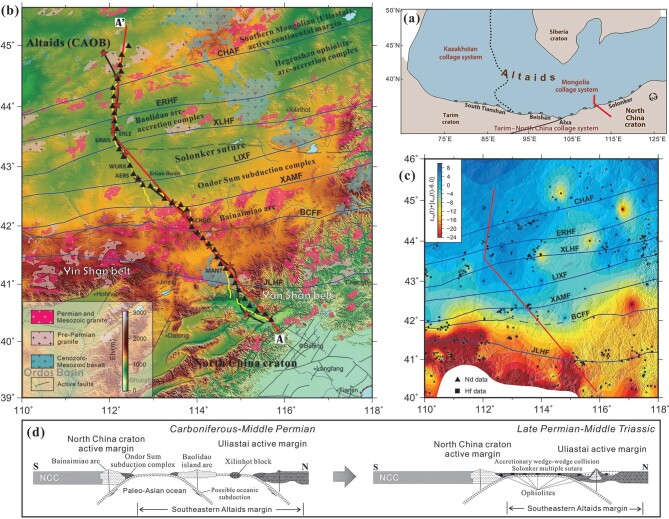
(a) Tectonic sketch map of the Altaids and the surrounding North China, Tarim and Siberia cratons (after Xiao *et al.* [20]), showing the location of our seismic line (red solid line, AA’ in panel (b). (b) Location of the broadband seismic array (black-filled triangles) on a simplified topographic–geologic map. The yellow solid line indicates the wide-angle seismic-reflection and -refraction (WAR/R) profile. The black solid line indicates the deep-seismic-reflection profile. Red-filled stars mark the locations of 12 large shots. Red solid line AA’ indicates the reference profile used for projection. JLHF, Jining–Longhua fault; BCFF, Bayan Obo–Chifeng fault; XAMF, Xar Moron fault; LIXF, Linxi fault; XLHF, Xilinhot fault; ERHF, Erenhot fault; CHAF, Chagan Obo fault. (c) Nd + Hf isotopic map of the NCC–Altaids transition. Isotopic data were extracted from Wang *et al.* [6] with 6.0 subtracted from the values of the Hf data before combination with the Nd data. The red solid line indicates our seismic line AA’ in panel (b). (d) A diagram illustrating the construction of the southeastern Altaids margin from amalgamation with the northern NCC during the late Paleozoic to Mesozoic time after the Paleo-Asian ocean closure (modified from Xiao *et al.* [8,20]).

Characterizing the growth and evolution of continental crusts in a precise way requires quantitative estimation of the crustal compositional architecture that reflects various degrees of crustal fractionation and maturation [1,2]. This bears great importance not only for deepening our understanding of the continental-evolution mechanism, but also for resource explorations because the phase of crustal growth proves to be further linked to large-scale mineralizations (e.g. [3,4]). The North China Craton (NCC) juxtaposed with the Altaid accretionary orogen (Fig. 1) involves a complex tectonic transition on its northern margin and has undergone several major episodes of crustal growth and evolution including the early-Precambrian cratonization and the Paleo-to-Mesozoic lithospheric reworking and modification whereas the Altaids (or Central Asian Orogenic Belt) preserves the largest Phanerozoic juvenile crustal volume on Earth [3,5,6]. Therefore, the northern-NCC-to-southeastern-Altaids transition may serve as an ideal laboratory for unraveling the mechanisms and characteristics of the compositional evolution of continental crusts subject to various evolutional phases. Geochemical and isotopic data have been typically used as an ideal proxy to quantify the degree of compositional maturation of continental crusts and a growing number of isotopic mapping efforts have been constructed aiming to trace the nature of the basement rocks of the NCC and the Altaids (e.g. [5,6] and references therein). Based on the apparent terrane difference shown by the isotope data, some recent work [6,7] revealed the contrasting nature and ages of the continental crust basements on the two sides of the Solonker suture (Fig. 1c)—an important boundary between the southeastern Altaids and the NCC. The Solonker suture was identified by using surface-geological observations as marking the final closure of the Paleo-Asian ocean in the end Permian to middle Triassic [3,8,9]. Previous geophysical observations conducted across the northern-NCC-to-southeastern-Altaids transition (hereafter termed as the NCC–Altaids transition involving the Solonker suture and the adjacent terranes on both sides; Fig. 1b) were mostly aimed at tracing the fossil subduction zones of the Paleo-Asian ocean plate preserved in the crust and upper mantle (e.g. [10–13]). However, in addition to the bulk nature of the continental basements reflected by the isotopic data, more precise and delicate proxies are still needed to constrain the compositional architecture, i.e. rock lithology (composition) and corresponding structural variation, in the crust across the NCC–Altaids transition, which may have recourse to the geophysical properties. This would therefore enable us to achieve a thorough comprehension of the Phanerozoic crustal evolution of the complex margin of the NCC and its relationship with the neighboring Altaids.

Estimation of rock lithology (composition) in the deep crust has been largely relying on geophysical methods, especially wide-angle seismic-reflection and -refraction (WAR/R) profiling (e.g. [14]). The non-uniqueness in relating single parameters such as P-wave velocity to rock lithology largely hinders a precise interpretation of the geophysical data, which can be relieved by using combined constraints of multiple geophysical parameters. Compared with single (compressional or shear) wave-speed constraints, the P-to-S wave-speed ratio Vp/Vs is a physical property that is more closely related to the rock composition and rheological state (partial melting, fluid) in the crust [2]. Combined constraints of Vp/Vs with other parameters such as Vp and density have proven superior in the estimation of crustal compositions (e.g. [15,16]). In this paper, based on a linear broadband seismic array that traverses the transition zone from the northern NCC margin to the southeastern Altaids (Fig. 1b), we conducted Vs and Vp/Vs imaging, combined with a-priori Vp data, to jointly constrain the crustal composition and structure across the NCC–Altaids transition. When integrated with the collinear deep-seismic-reflection profile and informed by isotopic mapping, our results provide new insights into the tectonic regime and crustal evolution of the NCC–Altaids transition.

## TECTONIC SETTING

The NCC has an Archean-to-Paleoproterozoic crustal basement that was cratonized at ∼1.85 Ga and subsequently covered by a thick Proterozoic–Paleozoic sedimentary sequence [17,18]. However, the NCC has undergone the Permian to early-Triassic Paleo-Asian ocean closure and the Paleo–Pacific plate subduction since the Triassic in succession. The cumulative influence of the preceding two magnificent tectonic events might have been concentrated on the northeastern NCC and led to large-scale and considerable lithospheric reworking of the northeastern part of the NCC, particularly during the Mesozoic—a time of decratonization for the eastern NCC [18].

The convergence of the Mongolian collage system in the north and the North China Craton in the south (Fig. 1a) lasted during most of the Paleozoic time. The eastern Paleo-Asian ocean in between continued its closure processes through possible divergent double subduction [19,20]. Multiple micro terranes including several island or continental arcs and subduction complexes developed in the Paleo-Asian ocean during the early- to middle-Permian construction of the southeastern margin of the Altaids (Fig. 1d). However, this tectonic belt evolved into an amalgamation of two active continental margins of Southern Mongolia (Uliastai) in the north and the NCC in the south during the final closure of the eastern Paleo-Asian ocean along the Solonker suture in the late Permian to middle Triassic. Soft collision of the two subduction accretionary wedges generated the continental crust of the Solonker multiple suture (constituting the southeastern Altaids margin) as a wide-range multiple-terrane accretionary belt, as evidenced by the scattered ophiolitic mélanges and accretionary complexes distributed within this belt [20] (Fig. 1d). The above tectonic evolution of the eastern Paleo-Asian ocean characterizes the southeastern Altaids as a typical accretionary orogen with a predominant Phanerozoic juvenile crustal basement [3,9]. The present-day specific Solonker suture is marked by a narrow belt bounded by the Xilinhot and Linxi faults, and is juxtaposed with the Baolidao arc-accretion complex belt in the north and the Ondor Sum subduction complex belt in the south (Fig. 1). Structural mapping suggests that the Linxi fault and the Jining–Longhua fault, which are two major boundary faults on the surface [8,21], dip to the north in the shallow level.

## RESULTS

**Figure 2.**
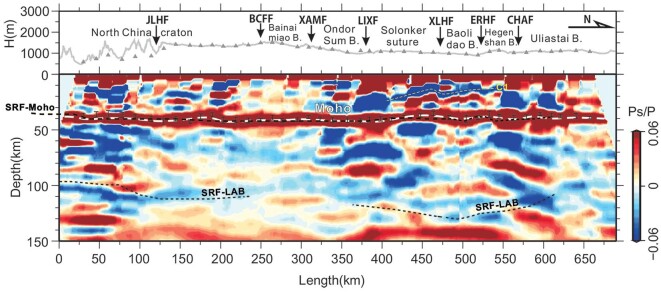
P-wave receiver-function image from the broadband seismic array along AA’, showing the crustal and upper-mantle structure across the NCC–Altaids transition. Topography and station positions are shown on the top. The Moho interface is marked using a thick-dashed line, with error bars superposed indicating the Moho depths derived from the H-*κ* stacking. Moho and LAB interfaces from our previous S-wave receiver-function (SRF) recognition [12] are shown here for comparisons. B, belt. For abbreviations of faults, see Fig. 1.

**Figure 3.**
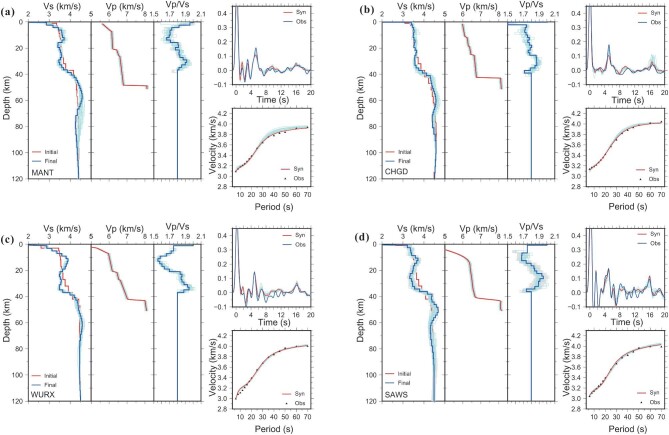
Results of the joint inversion of receiver functions and dispersions with Vp constraints at four seismic stations (locations denoted in Fig. 1b). For each station, the left panel shows the inverted best-fitting Vs model (blue line, left), the input Vp model (middle) and the resulting Vp/Vs model (blue line, right) from the joint inversion; the upper-right panel shows the fit between the observed (blue) and the predicted (red, synthetic from the best-fitting model) receiver functions; lower-right panel shows the fit between the observed (black triangles) and the predicted Rayleigh wave-phase dispersions (red line). The 5000 best models are plotted to show the uncertainties of the Vs and Vp/Vs models (gray corridors). Cyan thin lines show perturbation tests regarding the effects of the input Vp model uncertainties on the joint inversion results. A random velocity perturbation within ±0.1 km/s was added to the input Vp model before inversion and repeated tests were performed 20 times.

Our linear broadband seismic array is ∼650 km in length and composed of 41 stations (continuously operating from October 2012 to April 2015) with an average interval of ∼15 km, traversing the transition zone from the northern NCC margin to the southeastern Altaids (Fig. 1). We processed the data using both receiver-function (RF) imaging and Rayleigh wave tomography (Fig. 2 and Figs S1 and S2); we then conducted joint inversion of receiver functions and surface-wave dispersions with a-priori P-wave velocity (Vp) constraints in the crust (Fig. 3). By virtue of introducing a Vp model (from WAR/R profiling) in the joint inversion as an independent constraint, the joint inversion scheme further reduces parameter ambiguities within the inversion, and simultaneously provide Vs and Vp/Vs structures of the crust and uppermost mantle (Fig. S3 and Fig. 3; [22]).

### RF image of the lithospheric structure

**Figure 4.**
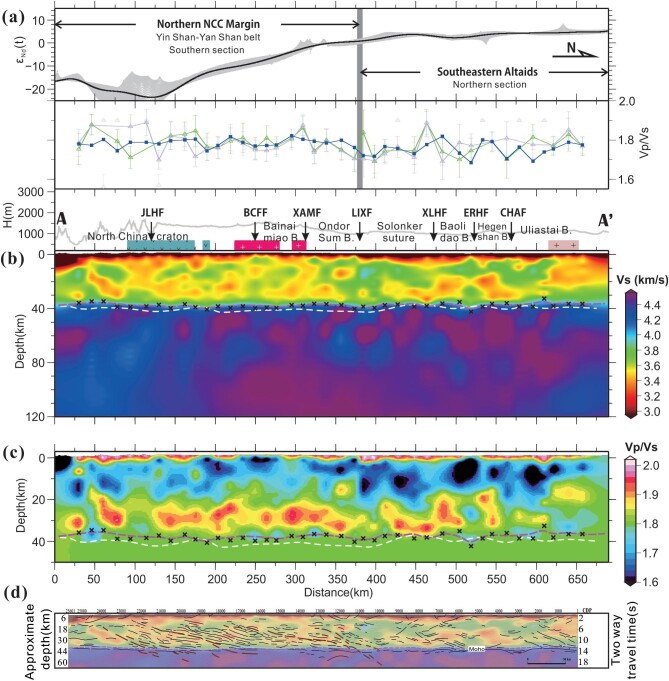
Vs and Vp/Vs models from joint inversion. (a) Upper panel: Nd + Hf isotopic data profile extracted from the isotopic map (Fig. 1c) along AA’. The gray corridor consists of data points within a 40-km-wide swath. The black curve is the result of fitting a polynomial regression model using the least-squares method. Lower panel: average Vp/Vs values of the sub-sediment crust at each station calculated from the inverted Vp/Vs model in panel (c) (blue-filled squares) and from an improved H-*κ* stacking method by Yu *et al.* [54] (green and light-blue triangles). Green triangles show H-*κ* results using full-back-azimuth (360°) receiver-function data while light-blue triangles show those using receiver-function data within the southeastern back-azimuth (90–180°). Error bar: 1 σ (standard deviation). The outlier data beyond the preset range (1.6–1.9) were excluded (gray triangles). (b) Vs section image along profile AA’. The white dashed line is the Moho interface defined by the CCP receiver-function image in Fig. 2, while the magenta dashed line indicates the Moho interface traced on the inverted Vs image along the top interface of the ∼4.0-km/s Vs zone. Black markers ‘×’ indicate Moho depths identified as locations of the maximum gradients of Vs increasing with depths in the lowermost crust beneath each station. For surface geology, see Fig. 1b. (c) Vp/Vs section image along profile AA’. Symbols denoting the Moho are duplicates of those in panel (b). (d) Superimposition of our inverted Vs model on a collinear deep-seismic-reflection profile (Tan *et al.*, 2024, unpublished data) with the line drawings of high-amplitude reflectors highlighted (colored and black dashed lines). The magenta dashed line indicates the Moho interface identified from the Vs model (panel (b)).

The migrated common-conversion-point (CCP) image of the receiver functions presents a strong positive-polarity conversion at the crust–mantle boundary (Moho) that runs in a flat pattern at a depth of ∼40 km along the whole profile (Fig. 2). A minor (∼5 km) bulge of the Moho occurs beneath the Solonker suture zone in ∼400 to 500-km distance section. The Moho interface constrained by the CCP image is generally consistent with those constrained by the H-*κ* grid search (Fig. S4) and joint inversion methods (Figs 2 and 4). The mantle part of the RF image displays abundant negative-polarity phases, among which those appearing in the depth range of ∼100–120 km may be correlative to the lithosphere–asthenosphere boundary (LAB) (Fig. 2; consistently with our previous S RF results [12]). An apparent feature is the contrast in the crustal structure across the Linxi fault at ∼375-km distance. The crust north of the Linxi fault is dominated by an inclined and faulted negative-polarity convertor (C1 marked by a thin dashed line in Fig. 2), while the crust south of this fault manifests as a subhorizontal pattern of the intracrustal convertors (see Fig. 2). Our data tests further confirm that convertor C1 is dominated by independent direct-converted waves rather than artificial multiple waves (Fig. S5).

### Vs and Vp/Vs models from the joint inversion

The joint inversion procedure provides the Vs and Vp/Vs section models of the crust and uppermost mantle along profile AA’ (Fig. 4). In correspondence with the RF image (Fig. 2), both the Vs and Vp/Vs images display a systematic and distinct variation in the crustal structure across the Linxi fault from south to north (Fig. 4b and c). South of the Linxi fault, a major part of the crust is occupied by regions with low Vs values of <3.5 km/s (≤35% of the crustal volume on the section from 0 to 375-km distance); north of this fault, the crust (particularly the middle-upper crust) is dominated by relatively high-Vs (>3.6 km/s) regions on the section from 375- to 625-km distance, and the crustal Vs decreases farther north beyond 625-km distance. Correspondingly, the Vp/Vs image (Fig. 4c) displays a homogeneous layering structure of the crust south of the Linxi fault, with upper (Vp/Vs < 1.75) and lower (Vp/Vs > 1.85) crustal layers that are ∼10–15 km thick extending horizontally in a relatively flat and smooth pattern, whereas, north of the Linxi fault, the upper crust with Vp/Vs < 1.75 thickens abruptly to ∼20 km in thickness within the section from 375- to 625-km distance, in accordance with the upper-crustal high-Vs (>3.6 km/s) layer (∼5–20 km depth) in this section (Fig. 4b and Fig. S6). The crustal Vp/Vs structure north of the Linxi fault manifests strong lateral heterogeneity in contrast to the counterpart south of this fault.

Another important feature is the distinct variation in the seismic property of the lithospheric mantle along profile AA’ (Fig. 4b). With the Jining–Longhua fault as a boundary, the uppermost mantle south of it displays low Vs of ∼4.0–4.2 km/s, while the uppermost mantle north of this boundary presents an overall high Vs of >4.4 km/s (Fig. 4b). This is consistent with our S RF identification of a step in the LAB interface underneath the Jining–Longhua fault (Fig. 2) [12].

### Validation assessment

We make quantitative assessments on the uncertainties of the resulting Vs and Vp/Vs models of each station site. The Vs models have converged well within the last 5000 searches and have a minor uncertainty of ±0.2 km/s (Fig. 3). The input Vp data from the WAR/R profiling typically have an uncertainty of 0.1 km/s [23] and thus we make perturbation tests concerning the input Vp model uncertainties through adding a random velocity perturbation of ±0.1 km/s before inversion and repeatedly testing 20 times. The final Vs models present uncertainties within a range of ±0.2 (km/s), while the final Vp/Vs models present uncertainties mostly within a range of ±0.05 that result from either the inversion or the input Vp data (Fig. 3). Additionally, the effect of uncertainties with input receiver functions and surface-wave dispersions has been estimated by previous researchers, who added 5% of white noise into both the observed receiver functions and the dispersions, and found that the resulting Vs and Vp/Vs models retained a stable pattern [24]. In general, the Vs and Vp/Vs models from the joint inversion are robust and stable, especially in retaining their patterns of variation with depth, even though we cannot achieve the precise values (particularly for the Vp/Vs). We also compare the crustal average Vp/Vs values at each station site calculated from the joint-inversion-derived Vp/Vs profile (Fig. 4c) with those derived from the H-*κ* stacking. The result displays a considerable degree of consistency between the two data sets (Fig. 4a), which further consolidates the validity of the resulting Vp/Vs models from joint inversions.

Here, we also conduct data tests and inspections to confirm that the systematic variation in the crustal Vs and Vp/Vs structures across the Linxi fault that is imaged by the joint inversion (Fig. 4) reflects real crustal structures rather than artifacts. A problem is whether or not the elevated upper-crustal Vs north of the Linxi fault (relative to that south of this fault), which is accordant with a thickened region in the upper crust with lowered Vp/Vs values (mostly ≤1.65) within 375- to 625-km distance (Fig. 4), is caused by the thickened sediments north of this fault as observed from the Vp profile (Fig. S3). We performed three aspects of data analysis to rule out the candidate for sedimentary artifacts during the data processing.

First, the forward modeling experiments confirm that adding a sedimentary layer on top of a homogeneous crust would not result in an artificial high-Vs (or low-Vp/Vs) layer in the crust after our joint inversion processing (Fig. S7a); simultaneously, a real upper-crustal high-Vs layer can be recovered successfully through joint inversion even with a relatively thick (e.g. 2.5-km) sedimentary layer on top of the crust (Fig. S7b). Second, our RF data analysis that combines systematic synthetic tests (aiming at the sedimentary and high-velocity-zone effects) with the observed RF waveforms (Fig. S5) further verifies the existence of two independent phases, C0 and C1, from two intracrustal interfaces that define an upper-crustal high-Vs layer north of the Linxi fault. Third, the different time–distance curves manifested by the direct-converted phases and the multiple-converted phases assist in discerning the independent phases from the interference of sediment-related reverberations (Fig. S5a and d; also seen in Zhang *et al.* [12]).

## DISCUSSION

### The crustal compositional variation across the NCC–Altaids transition

**Figure 5.**
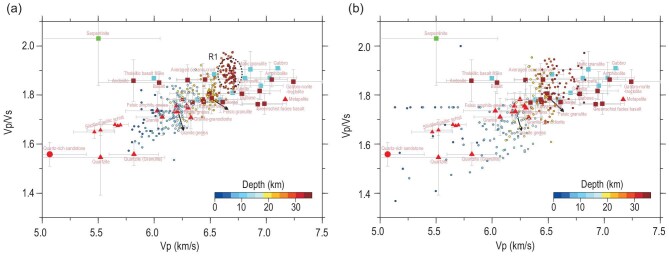
Vp/Vs versus Vp diagrams for crustal rocks (measured under the 600-Mpa condition; data collected from Christensen [2] and Kuo-Chen *et al.* [15]) with our Vp/Vs–Vp data of the southern section (a) and northern section (b) projected on top. Error bar: 1 σ (standard deviation). Red color-coded symbols: felsic rocks. Dark-red and cyan color-coded symbols: intermediate-mafic rocks. Green color-coded symbol: hydrated rock. Colors of the Vp/Vs–Vp data points denote depths.

The Vp/Vs–Vp diagrams have been used to efficiently estimate rock compositions through combining constraints from the Vp and Vp/Vs parameters (e.g. [14,15]). We project the Vp/Vs–Vp data of the southern and northern sections onto a Vp/Vs-versus-Vp diagram for crustal rocks that were measured by previous researchers in laboratories under a pressure condition of 600 Mpa (∼22-km depth) (Fig. 5). The distinct variation in the crustal seismic properties (Vs and Vp/Vs) and structures across the Linxi fault probably indicate a significant crustal compositional variation across this NCC–Altaids boundary. This is further illustrated by the Vp/Vs–Vp diagrams (Fig. 5), which show that the data-point distribution of the northern NCC margin (southern section) displays a compaction and sublinear pattern (Fig. 5a), while the data-point distribution of the southeastern Altaids (northern section) displays a diffuse pattern (Fig. 5b). The compaction/sublinear data-point distribution of the northern NCC margin is a manifestation of an evolved crust with layered homogeneous compositions, which is also reflected by the Vp/Vs profile (Fig. 4c). The upper, middle and lower crusts are characterized as felsic, intermediate and mafic rock materials, respectively, as indicated by the Vp/Vs–Vp diagram (Fig. 5a). In contrast, the diffuse data-point distribution of the southeastern Altaids is a manifestation of a structurally complex crust with heterogeneous and irregular layers of compositions. The felsic upper crust with Vp/Vs of <1.75 is significantly thickened, while the intermediate-to-mafic mid-lower crust with Vp/Vs of ≥1.75 also seems horizontally deformed, as shown by the Vp/Vs profile (Fig. 4c).

### The juvenile crust of the southeastern Altaids

The isotope map shows that the Linxi fault roughly divides the NCC–Altaids-transition crust into the primitive (i.e. juvenile, *ε_Nd_(t)* > 0) provinces in the north and the evolved (*ε_Nd_(t)* < 0) provinces in the south (Fig. 1b) according to the categories suggested by Wang *et al.* [6] regarding the isotopic provinces of the Altaids. The *ε_Nd_(t)* values on the northern section of our seismic profile are within a range of ∼0 to +6, corresponding to primitive (juvenile, *ε_Nd_(t)* = +4 to +6) and slightly primitive (*ε_Nd_(t)* = +4 to 0) crusts, whereas the *ε_Nd_(t)* values on the southern section are within a range of approximately –24 to 0, corresponding to slightly evolved (*ε_Nd_(t)* = –4 to 0), evolved (*ε_Nd_(t)* = –10 to –4) and highly evolved (*ε_Nd_(t)*<10) crusts (Fig. 4a) [6].

The characteristic of compositional and structural complexity in the crust of the northern section (Figs 4c and 5b) as analysed above may be a signature of the juvenile crust of the southeastern Altaids. A juvenile crust has essentially experienced an insufficient material differentiation process and retained compositional complexity [25–27]. The thickened felsic upper crust may have resulted from the massive stacking and imbrications of the newly differentiated intermediate-felsic upper-crustal materials after the early-Mesozoic wedge–wedge collision in the Solonker suture zone (e.g. [26,28,29]). A deep-seismic-reflection profile collinear with our broadband seismic array images a series of north- and south-dipping thrust faults in the crust beneath the southeastern Altaids, which are tectonically identified as compressional structures related to the pre-Jurassic Altaid orogen (Fig. 4d). Those thrust structures, including crustal-scale ductile shear zones and correlative fold-and-thrust systems comprising conjugate thrusts and imbrications (Fig. 4d; also interpreted by Zhang *et al.* [10]), coincide with and support our previous interpretation of the crustal complexity indicative of a juvenile crust of the southeastern Altaids.

Our RF imaging of the south-dipping interfaces in the crust beneath the Solonker suture (Fig. 2) is consistent with several previous magnetotelluric profiles that are subparallel to our observation line in this region and show a suite of south-dipping intracrustal conductors beneath the Solonker suture zone and its southern adjacent area [11]. A previous RF profile to the east of our seismic line also revealed a similar south-dipping intracrustal fabric beneath the southeastern Altaids [28]. We also see that the overall crustal frameworks as constrained by our RF profile and the collinear deep-seismic-reflection profile are very consistent, while the packages of north-dipping reflections in the deep level beneath the Solonker suture may represent strain fabrics that developed within the lower-crustal rocks and cannot be revealed by the broadband seismic imaging (Fig. 4d and Fig. 8). Our RF imaging, combined with the existing geophysical observations, supports the interpretation that the imaged intracrustal features beneath the Solonker suture probably indicate a fossil southward subduction of the Paleo-Asian ocean plate [11–13,28], which was proposed by previous geological observations [8].

### Partial reworking of the lithosphere beneath the northern NCC margin

The ancient crustal basement of the NCC is basically retained beneath its northern margin (the Yin Shan–Yan Shan belt), as indicated by the isotopic data (*ε_Nd_(t)* < 0) and our seismic recognition of the layered homogeneous compositions in the crust of this craton margin (Figs 1c, 4 and 5a). Both the isotopic and geophysical data suggest an evolved crust with long-time and high-degree compositional differentiations beneath the northern NCC margin, which are comparable with the Archean-Proterozoic crustal-basement components revealed by the isotopic mapping in the southeastern NCC [30].

However, intensive Mesozoic reworking of the NCC lithosphere, which manifested as multiple and alternate episodes of intraplate contractional deformation and tectonic extension in the northern NCC since the late Paleozoic, is documented in the Jurassic fold-and-thrust belts and the Permian-to-Mesozoic granitoids that are widespread in the Yin Shan–Yan Shan belt (Fig. 1b; [31,32]). Petrological studies have revealed that those Mesozoic granitoids were derived from multiple sources, including depleted mantle, enriched lithospheric mantle, ancient lower crust and juvenile crust, which suggests intensive crust–mantle interaction and significant continental crustal growth in the northern NCC margin during the Mesozoic [33–35].

**Figure 6.**
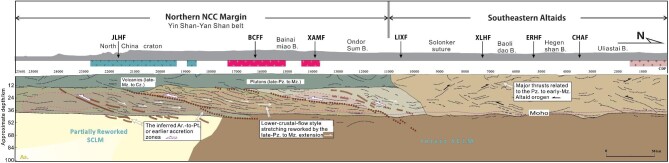
Conceptual interpretive model based on the Vs and Vp/Vs imaging combined with the deep-seismic-reflection profile. Purple dashed-line arrows mark the inferred Archean-to-Paleoproterozoic or earlier accretion zones (brown thick-dashed lines) fossilized in the lower crust of the northern NCC margin, which survived the Phanerozoic lithospheric reworking. The white arrows indicate lower-crustal-flow-style stretching formed during the late Paleozoic to Mesozoic extension. Black arrows indicate major thrusts correlative to the Paleozoic to early-Mesozoic Altaid orogen. The magenta-shaded region outlined with a pink dashed line indicates the lower-crustal low-Vs/high-Vp/Vs zone. As, asthenosphere; SCLM, subcontinental lithospheric mantle; Ar, Archean; Pt_1_, Paleoproterozoic; Pz, Paleozoic; Mz, Mesozoic. Cz, Cenozoic. The magenta dashed line indicates the Moho interface identified from the Vs model (see Fig. 4b). For the surface geology see Fig. 1b.

The crust–mantle interaction is accomplished through injection of the mantle-derived mafic magmas into the crust, triggering partial melting of the lower-crustal material and mixing with the crust-derived felsic magmas to different degrees (e.g. [36]). This process produces various hybrid magmas that are additions to the crust at various levels and change the crustal components of the northern NCC margin. Correspondingly, the isotope map shows *ε_Nd_(t)* values of greater than –10 in the northern part of the Yin Shan–Yan Shan belt (north of the Bayan Obo–Chifeng fault, Fig. 1c), which indicates some degree of reworking of the ancient crust beneath this craton margin. Therefore, here, we suggest that the intracrustal low-Vs and high-Vp/Vs zones observed beneath the Yin Shan–Yan Shan belt (Fig. 4)—such as the data points in the R1 region in Fig. 5a within a depth range of ∼28–35 km—probably represent the Mesozoic reworked or juvenile crustal material with changed components relative to the ancient metamorphic crustal basement of the NCC (Fig. 6). This interpretation is based on their high Vp/Vs values (∼1.85–1.90) and relatively low Vp values (∼6.6–6.75 km/s) shown by the data in R1, of which the Vp values are slightly higher than those of the average oceanic crust (∼6.55 km/s) but significantly lower than those of typical high-grade metamorphic rocks in the lower part of a highly differentiated cratonic crust (e.g. mafic granulite, amphibolite, >6.8 km/s, Fig. 5a).

The preceding Vp/Vs–Vp feature of the reworked/juvenile material recognized in R1 implies that the present lower crust of the northern NCC margin is mostly low-grade in metamorphism and may have evolved from mixing of the mantle-derived intrusions with the crustal materials during the Mesozoic time (Fig. 6). The ancient mafic lower crust might have been delaminated after high-pressure metamorphism during thickening and extension events in the Mesozoic. This inference is based on the present thin crust (∼40 km) with low velocity in the lower crust beneath the Yin Shan–Yan Shan belt (Fig. 4), combined with geochemical evidence for lower-crustal delamination derived from Mesozoic volcanic rocks in the eastern Yan Shan belt [37]. The authors identified that those Mesozoic magmas carry inherited Archean zircons and have Sr–Nd isotopic compositions overlapping those of eclogite xenoliths derived from the lower crust of the NCC, and thus suggested that the lavas derive from ancient mafic lower-crustal materials that were delaminated and subsequently melted and interacted with mantle peridotite [37]. The low shear velocity characteristic of the uppermost mantle in the ∼0 to 180-km section suggests that intense lithospheric reworking and destruction in the late Mesozoic to Cenozoic time were limited to the southern end of this section, particularly south of the Jining–Longhua fault. Our own and other previous S-wave RF images also revealed that the LAB deepens abruptly from <100 to >110 km in depth across the Jining–Longhua fault from south to north (Fig. 2; [12,38]).

### Implications from combination with deep-seismic-reflection profiling

We superimposed a previously conducted collinear deep-seismic-reflection profile that we reprocessed and reinterpreted recently onto our Vs model (Fig. 4d). First, the reflection profile presents dramatically contrasting crustal structures between its northern and southern sections, roughly divided by the Linxi fault. This is markedly consistent with the underlying crustal Vs structural signatures that highlight the north–south contrast, as discussed above (Fig. 4d). The southern section of the reflection profile displays crustal layering with a highly reflective lower crust and a non-reflective upper crust, whereas the northern section manifests a diffuse and less reflective crust. In the lower crust of the southern section, packages of laterally continuous and shallowly dipping reflections are bounded by four major reflectors (brown thick-dashed lines in Figs 4d and 6). Those reflections exhibit a low-angle thrust–nappe geometry, but flatten out into subhorizontal reflectivity in the lowermost crust while the major reflectors can be extended down into the uppermost mantle through several Moho interruptions. Zhang *et al.* [10] previously interpreted these major deep reflectors as a post-collisional south-directed crustal thrust system after the Paleo-Asian ocean closure that is similar to that of the modern Himalayas; the interpretation was based on their observation and inference that the upward projections of these large-scale deep reflectors could be connected directly to the surface fault traces, cutting through the older (Paleozoic) granitic bodies in the upper crust. Alternatively, here, we prefer to interpret the lower-crustal framework of the southern section as representing slivers of subducted and imbricated Precambrian crustal terranes (Fig. 6)—possible relics of the plate subductions operating in the northern NCC during the Paleoproterozoic time (e.g. [39]). This interpretation is based on (i) the structural architecture of unconformity between the lower-crustal thrust system and the overlying upper-crustal strata and plutons, which is corroborated by the new recognition (from the reprocessed deep-reflection profile) of the terminations of these deep-dipping reflectors at the base of the upper crust (∼12–15 km in depth) (Figs 4d and 6); and (ii) the close resemblance of the shallowly dipping deep reflectors to that revealed in the lower crust of the Archean western superior province of Canada, which defines a large lower-crustal accreted slab from the Archean subduction [40].

Second, it is remarkable and interesting that the abundant shallowly dipping reflections spatially coincide with the low-Vs/high-Vp/Vs zone in the lower crust beneath the Yin Shan–Yan Shan belt, because the lower-crustal reworking suggested by the low-Vs/high-Vp/Vs zone as discussed above should have destructed the ancient structures. It is likely that the multiple episodes of the late Paleozoic to Mesozoic extensions had caused continental spreading that could use these existing ancient thrust-imbrication structures through developing them into extensional shear zones (e.g. [41,42]). Pervasive later high-temperature melts may then have risen along those shear zones, changed the lower-crustal components through mixing and re-weakened the lower crust. This process retained the ancient low-angle structures probably by exploiting the existing gneissic foliations and simultaneously resulted in the low-Vs/high-Vp/Vs zone in the lower crust (e.g. [43]). Considerable rising and addition of the melts through the sheeted lower crust into the upper crust is accordant with the late Paleozoic and Mesozoic granitic plutons and volcanics that are widespread at the surface of the Yin Shan–Yan Shan belt and correlate with the non-reflective upper crust (Fig. 6). Similar continental spreading processes involving lower-crustal flow (stretching) during tectonic extension were imaged by seismic-reflection profiling as pervasive lower-crustal low-angle strain fabrics beneath the Yilgarn craton of southwest Australia [42] and were modeled by numerical experiments for post-collisional tectonics such as the South Tibetan detachment shear system (e.g. [44]).

## CONCLUSIONS

Our observation when integrating multiple seismic data sets and parameters identifies a significant variation in crustal composition and structure across the North China–Altaids transition. The Yin Shan–Yan Shan belt is recognized as the northern NCC margin and characterized by layered homogeneous compositions with signatures that point to an evolved crust, yet having undergone considerable reworking through lower-crustal-stretching-assisted melt migration and mixing since the late Paleozoic to Mesozoic eras. The northern NCC margin thus possesses a compositionally modified ancient crustal basement whereas the southeastern Altaids domain manifests crustal complexity in compositions and structures inferred to be indicative of the juvenile crust of a Phanerozoic accretionary orogen. Our results provide a successful case of imaging and integrating fine-scale deep physical properties to constrain the phase of crustal growth and evolution of continents.

## DATA AND METHODS

We extracted the P-wave receiver functions from selected teleseismic events (Ms ≥ 5.5) within an epicentral distance range of 30°–95° through deconvolution of the vertical components by the radial components under an epicenter-to-station radial-transverse (R-T) coordinate system (e.g. [45]). The procedure produced a total of 3497 high-quality receiver functions after further visual inspection (Fig. S3). We then constructed the CCP image by projecting every amplitude of the receiver functions into a 3D space domain through ray tracing based on a layered reference velocity model and stacking the amplitudes within designed bins onto a designated cross section (Fig. 2). The stacking bins were set as 2 km in length along profile AA’ at the surface that expanded downwards according to the Fresnel zone and 0.5 km in depth. The reference velocity model is the IASP91 earth model with its crustal part modified by adopting a laterally variable crustal Vp/Vs ratio model from the H-*κ* stacking results (Fig. S4) and a collinear crustal Vp model (Fig. S3c) from an active-source WAR/R profile (Fig. 1b).

We utilized continuous vertical-component seismograms recorded at 126 broadband seismic stations, including 85 permanent stations from China Earthquake Administration (Figs S1 and S2) from March 2013 to April 2015 to conduct inter-station ambient noise cross-correlations. This was done following the procedure outlined by Bensen *et al.* [46]. First, the continuous ambient noise records were divided into 1-day segments and resampled at a frequency of 1 Hz. Subsequently, the instrument responses, trends and mean values were eliminated and a band-pass filter ranging from 6 to 40 s was applied to all 1-day segments. Then, to mitigate the impact of non-stationary noise sources and broaden the bandwidth of ambient noise records, temporal normalization and spectral whitening were performed. Finally, the 1-day segments between each pair of seismic stations were cross-correlated and linearly stacked to obtain the final cross-correlations. An example of the record sections of vertical–vertical cross-correlations between station AERS (location denoted in Fig. 1b) and all the other stations, covering periods ranging from 6 to 40 s, is shown in Fig. S1. Notably, distinct fundamental Rayleigh wave signals are discernible in both the causal and acausal portions of each cross-correlation.

We employed the two-plane wave tomography method [47] to determine the phase velocity of teleseismic Rayleigh waves with periods ranging from 25 to 100 s. This method accounts for the incoming non-planar wave field caused by lateral heterogeneities in Earth's structures. A total of 571 earthquakes with magnitudes of >6.0 and epicentral distances ranging from 30° to 120° were selected. The fundamental mode Rayleigh waves were isolated from the vertical components at 11 selected periods (25–100 s) using a zero-phase-shift band-pass Butterworth filter. Only seismograms with an SNR (signal-to-noise ratio) of >15 and recorded by >20 stations were included in the tomography analysis [48]. The study area was parameterized using a 2D spatial grid with a node spacing of 0.5° in both longitude and latitude. The broadband dispersion curves were obtained at each 0.5° × 0.5° grid node with periods ranging from 6 to 100 s by extracting phase velocities from ambient noise tomography and teleseismic surface-wave tomography methods (Fig. S2). For the overlapping periods of 25–40 s, a weighted average of the phase velocities was calculated based on the uncertainties of both data sets.

We assembled three independent seismic data sets along profile AA’ (Fig. S3): the RF and dispersion data as derived from preceding procedures, coupled with crustal Vp data measured from an active-source WAR/R profile that we conducted previously in 2009–2010 [23,49] and extends exactly along the same line as the broadband seismic profile AA’ (Fig. 1b). The WAR/R profile collected seismic data from 12 large shots with 500–1500 kg of explosive charges and the seismic stations were spaced at intervals of 1–3 km. Typically, the joint inversion of receiver functions and surface-wave dispersions can reduce the parameter ambiguities within the inversion [50]. However, Li *et al.* [22] demonstrated that the conventional application of a fixed Vp/Vs value (e.g. the global average of 1.75) in the joint inversion could generate a less accurate or even wrong resultant Vs model if the applied Vp/Vs deviates greatly from the actual value. Following the scheme illustrated in Li *et al.* [22], we introduce an a-priori Vp model (WAR/R profiling) in the joint inversion as an independent constraint that unfixes the Vp/Vs in the crust and the inverted results simultaneously provide Vs and Vp/Vs structures of the crust and uppermost mantle (e.g. [51,52]). We use the Vs model from sole inversion of the surface-wave dispersions as the initial model of the subsequent joint inversion (see Fig. 3). The layered crustal Vp/Vs is determined by the Vs from inversion and the interpolated Vp at the same depth. A separate linear gradient is employed at the surface to characterize the sedimentary layer, which can significantly improve the RF wave fitting. We fixed the Vp/Vs for the sedimentary layer as 2.0 [53] and searched for the optimal thickness and top/bottom velocities in a designated range (herein, 0 ≤ *H_sedi_* ≤ 7 km, 0 ≤ *Vs_sedi_*___*_top_* ≤ 1.0 km/s and 1.0 km/s ≤ *Vs_sedi_*___*_bottom_* ≤ 3.0 km/s). The upper-mantle Vp/Vs is set as a global average of 1.8.

## Supplementary Material

nwae171_Supplemental_File
