# Supplementary material for: Constraints on crustal compositional architecture across the North China–Altaids transition and implications for craton margin reworking
Source: Natl Sci Rev. 2024 May 15;11(5):nwae171. doi: 10.1093/nsr/nwae171 (PMC11160325; doi:10.1093/nsr/nwae171)
Supplement: nwae171_Supplemental_File [file nwae171_supplemental_file.doc]

**Supplemental Material for**

**Seismic constraints of crustal compositional architecture across the North China-Altaids transition and implications for craton margin reworking**

Zhuo Ye^1,2*^, Xiaomiao Tan^3*^, Rui Gao^3*^, Qiusheng Li^2^, Hongshuang Zhang^2^, Xiaoyang Wu^4^, Wenhui Li^2^, Yingkang Li^5^

*^1^SinoProbe Center, Chinese Academy of Geological Sciences, Beijing 100037, China*

*^2^Key Laboratory of Deep-Earth Dynamics, Ministry of Natural Resources, Institute of Geology, Chinese Academy of Geological Sciences, Beijing 100037, China*

*^3^School of Earth Sciences and Engineering, Sun Yat-sen University, Guangzhou 510275, China*

*^4^Key Laboratory of Marine Geology and Environment, Institute of Oceanology, Chinese Academy of Sciences, Qingdao 266071, China*

*^5^China Geological Sample Information Center, MLR, Yanjiao 065201, China*

***** Corresponding authors: Z. Ye ([yezhuo5000@hotmail.com](mailto:yezhuo5000@hotmail.com)), X. Tan (tanxiaomiao@163.com), and R. Gao (gaorui66@mail.sysu.edu.cn)

**
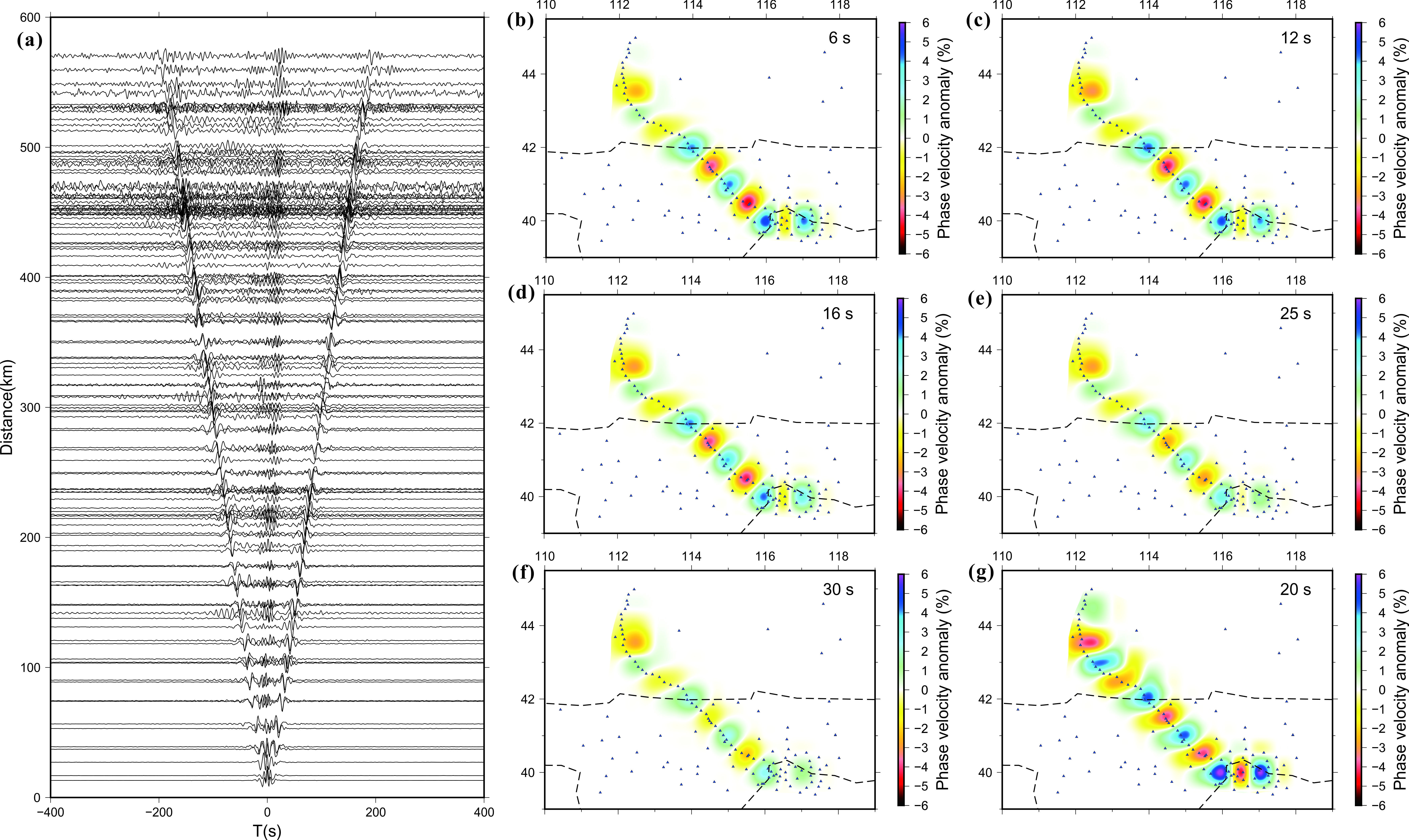
**

**Fig. S1.** (a) Vertical-vertical cross-correlations between station AERS and all the other stations. (b-f) Sensitivity test showing recovered models along the broadband seismic profile for selected periods. (g) Input model shown at the period of 20 s. Note the pattern of the input is generally recovered well along the profile, while the amplitude recovery is around 60% - 80% level.


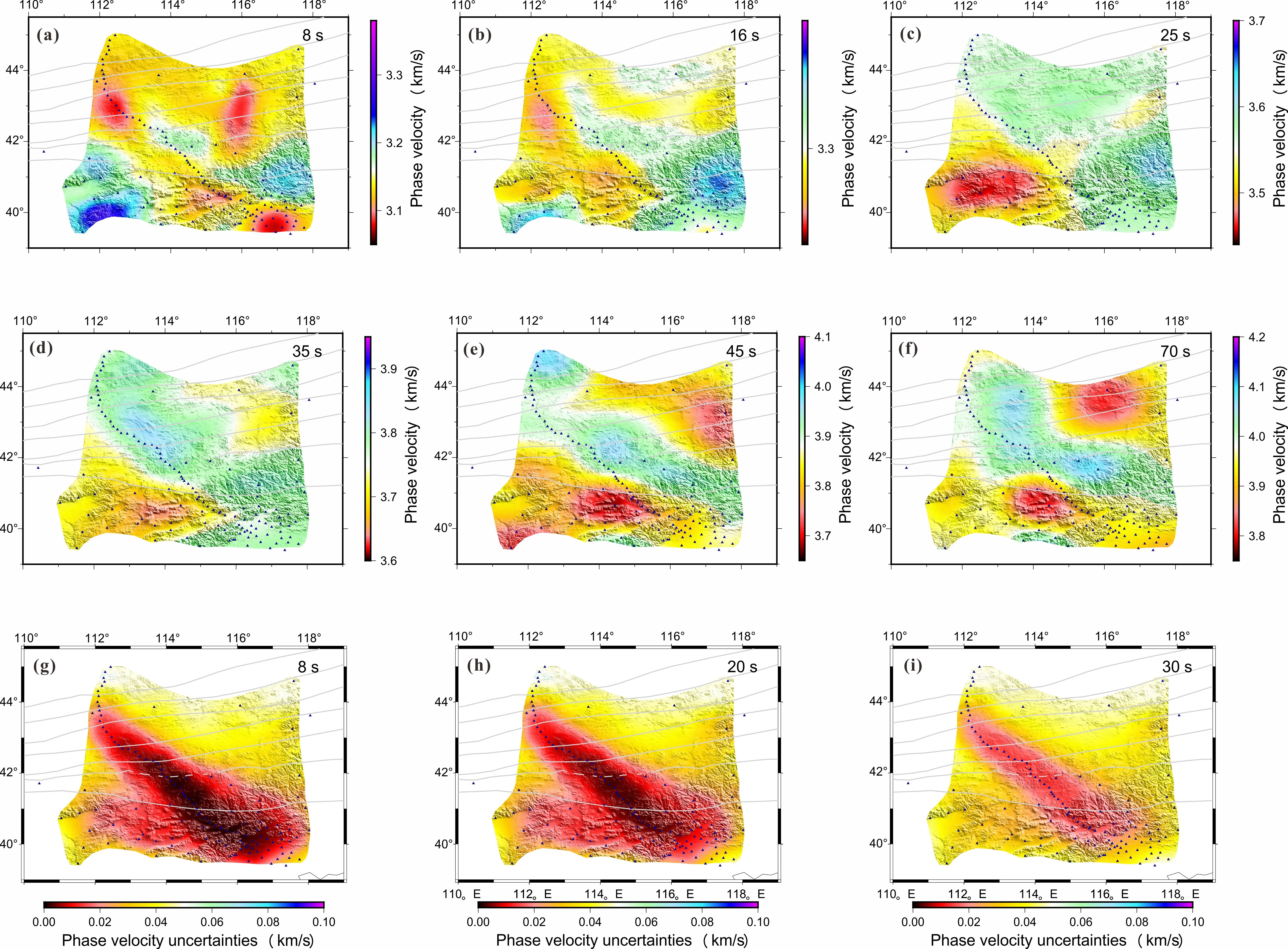


**Fig. S2.** Rayleigh wave phase velocity maps (a-f) and the corresponding uncertainties (g-i) for selected periods.

**
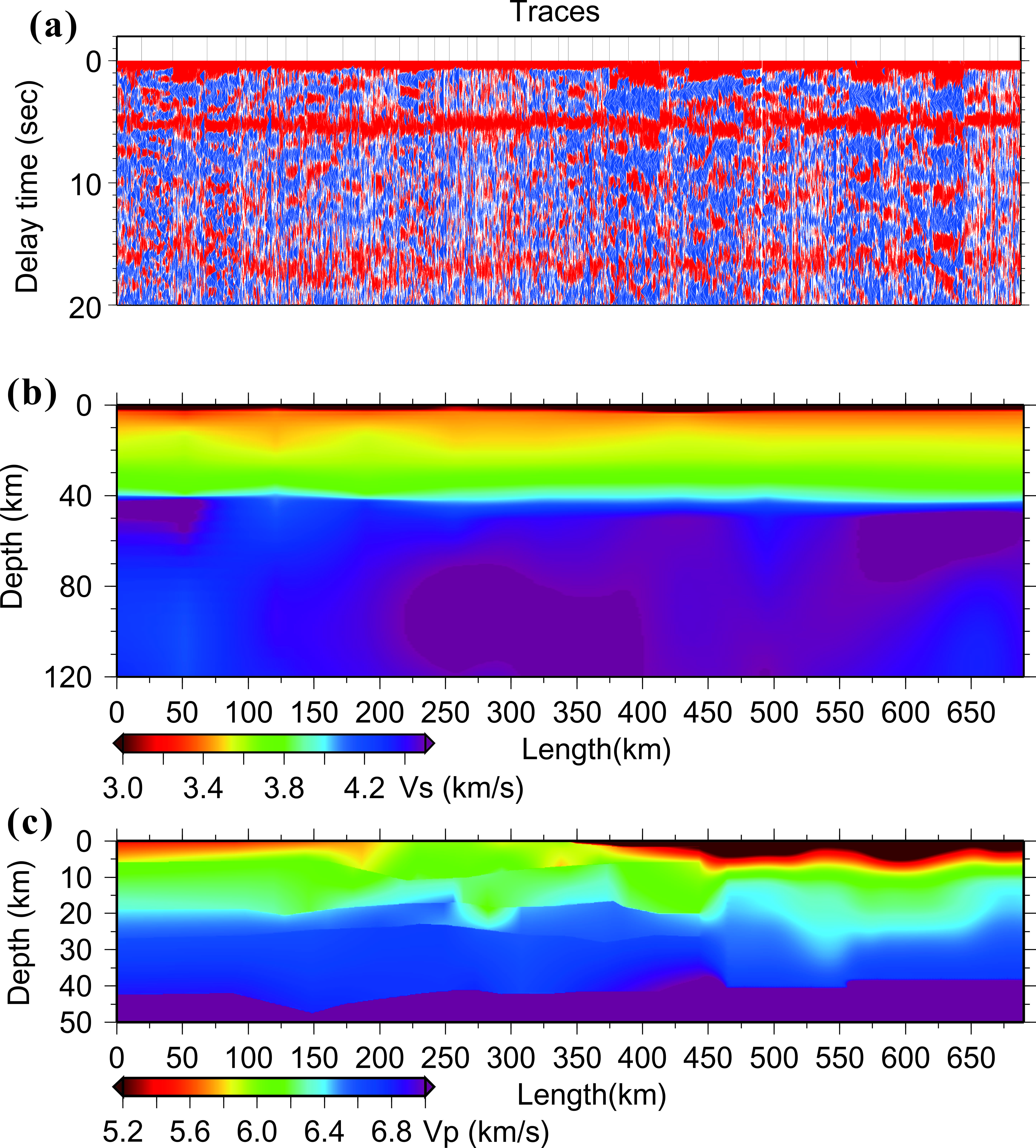
**

**Fig. S3.** Initial datasets used for joint inversion of receiver functions (RFs) and surface-wave dispersions with Vp constraints (along profile AA’, see Fig. 1b for location). (a) Trace section of raw RFs windowed between 0-20 s after P arrivals from the broadband seismic array. RF traces for each station are sorted by the angles (0-180°) between the back-azimuthal radial line of each RF ray and the profile orientation (N319.9°E, N8.1°E), in descending order. Red and blue colors shade the positive and negative amplitudes, respectively. The Gaussian filter parameter is set as 2.5 with a high cut-oﬀ frequency of ~1.2 Hz. RFs of each station were stacked in every 5° back-azimuths and 5° great-circle epicentral distances. (b) Vs profile from surface-wave dispersions. (c) Vp profile from our previously conducted wide-angle seismic reflection and refraction (WAR/R) profile [1,2].

**
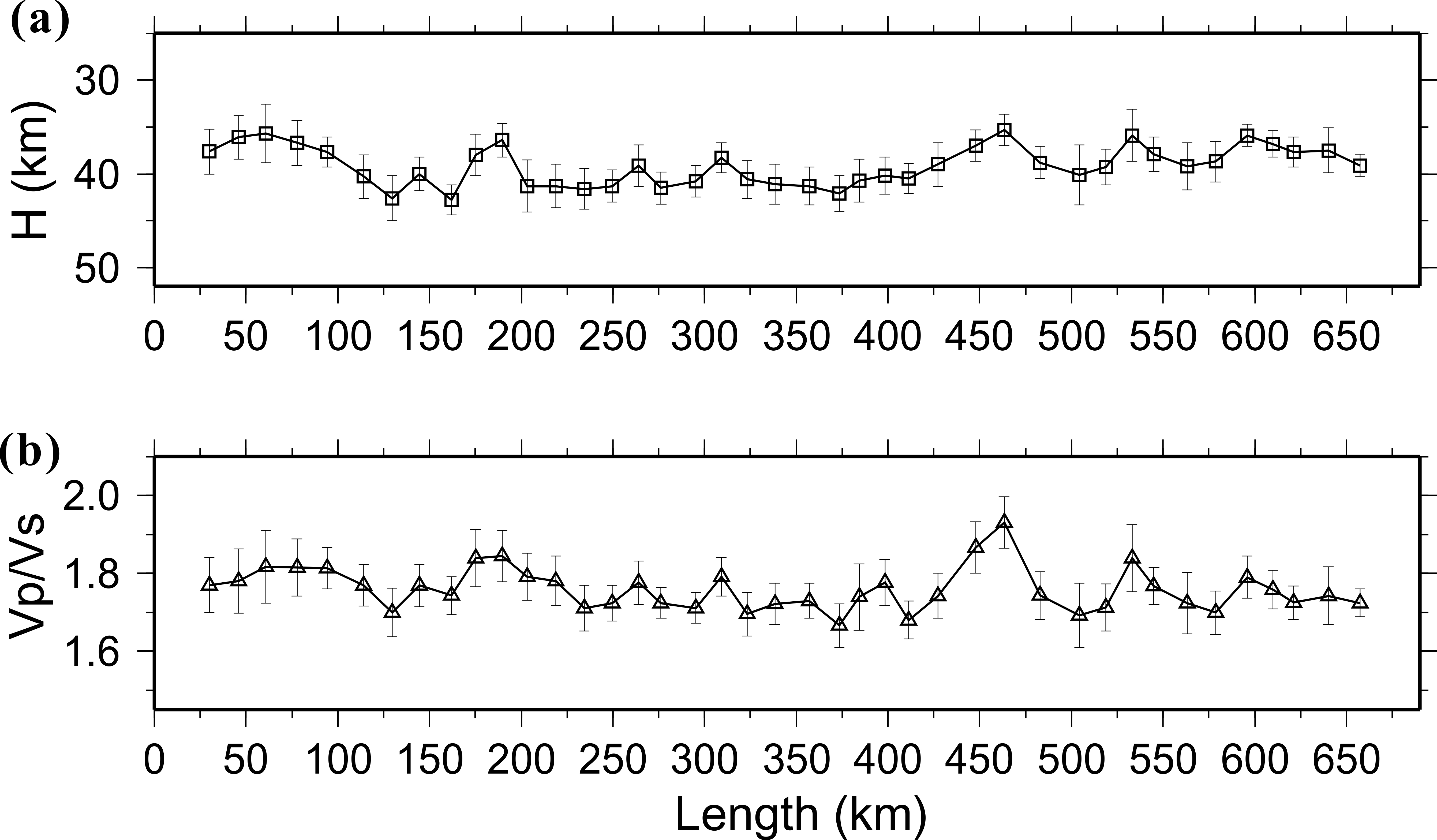
**

**Fig. S4.** Bulk crustal thicknesses (a) and Vp/Vs ratios (b) along profile AA’ estimated from the H-*κ* stacking method [3]. Error bar: 1 $\sigma$ (standard deviation).

**
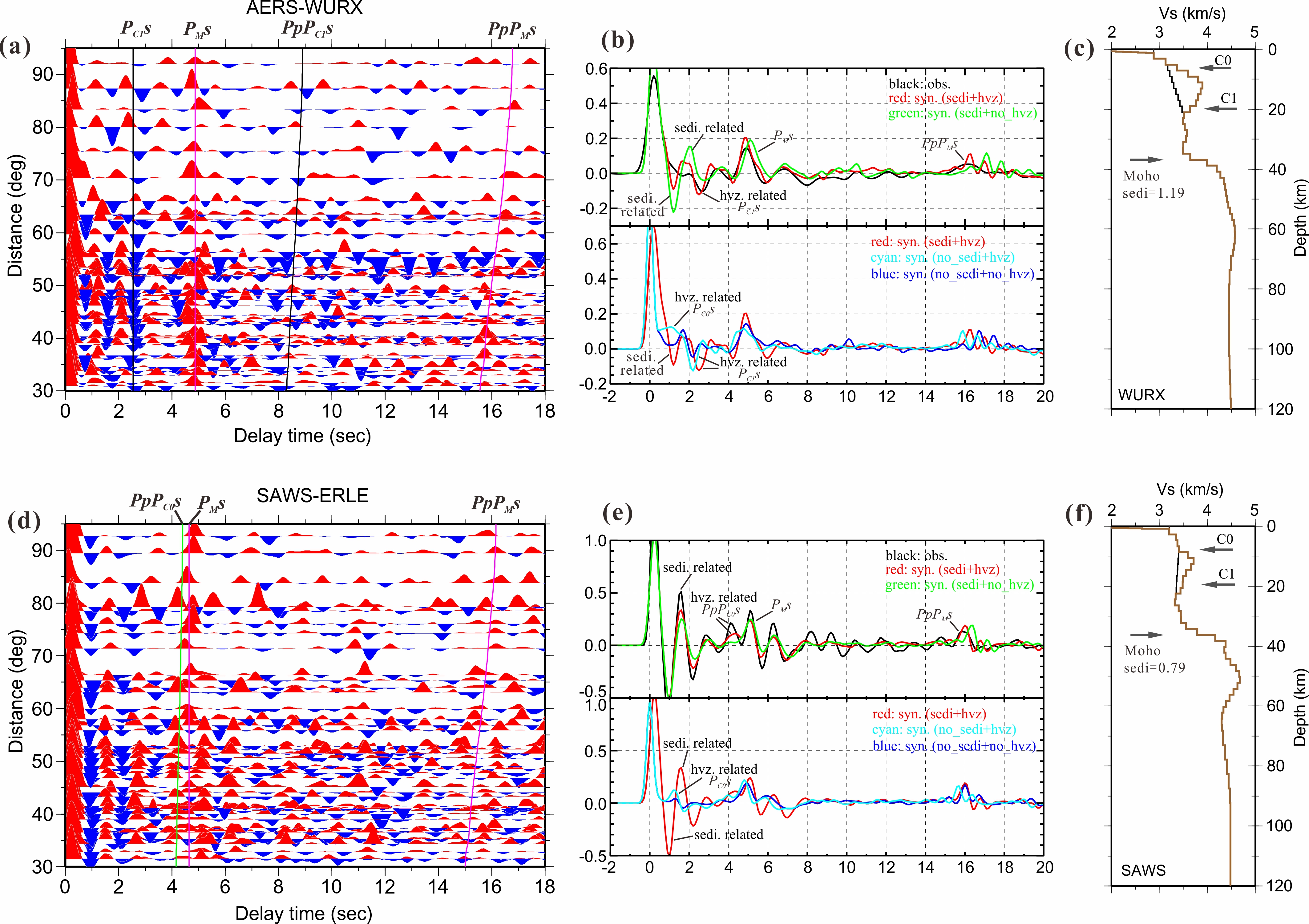
**

**Fig. S5.** RF trace sections of neighboring stations AERS and WURX (a) and neighboring stations SAWS and ERLE (d) (for station locations see Fig. 1b). RFs were bin-stacked and aligned according to epicentral distances in every 3° step. Black lines in (a) are synthetic phases denoting the direct converted wave $P_{C1}s$from an intracrustal (C1) interface and its first multiple wave $PpP_{C1}s$ (model parameters are Vs=3.45 km/s, Vp/Vs=1.75, and $H_{IC}$=19.8 km). Green line in (d) is a synthetic phase denoting the first multiple wave $PpP_{C0}s$ from an intracrustal (C0) interface (model parameters are Vs=3.2 km/s, Vp/Vs=1.72, and $H_{IC}$=9.0 km). Magenta lines are synthetic phases denoting the direct converted wave $P_{M}s$ from the Moho and its first multiple wave $PpP_{M}s$ (model parameters are Vs=3.55 km/s, Vp/Vs=1.76 (WURX) or 1.75 (SAWS), and $H_{Moho}$=38.5 km (WURX) or 37 km (SAWS)). (b) and (e) show synthetic tests for confirming the presence of two intracrustal interfaces C0 and C1 that define an upper-crustal high-velocity zone (HVZ) under thick sediments. We removed the upper-crustal HVZ or the sediment layer or them both in sequence from the inverted Vs models (black lines in panels c and f) and conducted comparisons between the corresponding synthetic RFs and the observed RFs to differentiate the HVZ-related convertors from the sediment-related artificial waves (panels b and e). We find that the direct converted wave $P_{C0}s$ is contaminated by the sediment-related reverberations and is not visible, but its first multiple wave $PpP_{C0}s$ can be identified in (e).

**
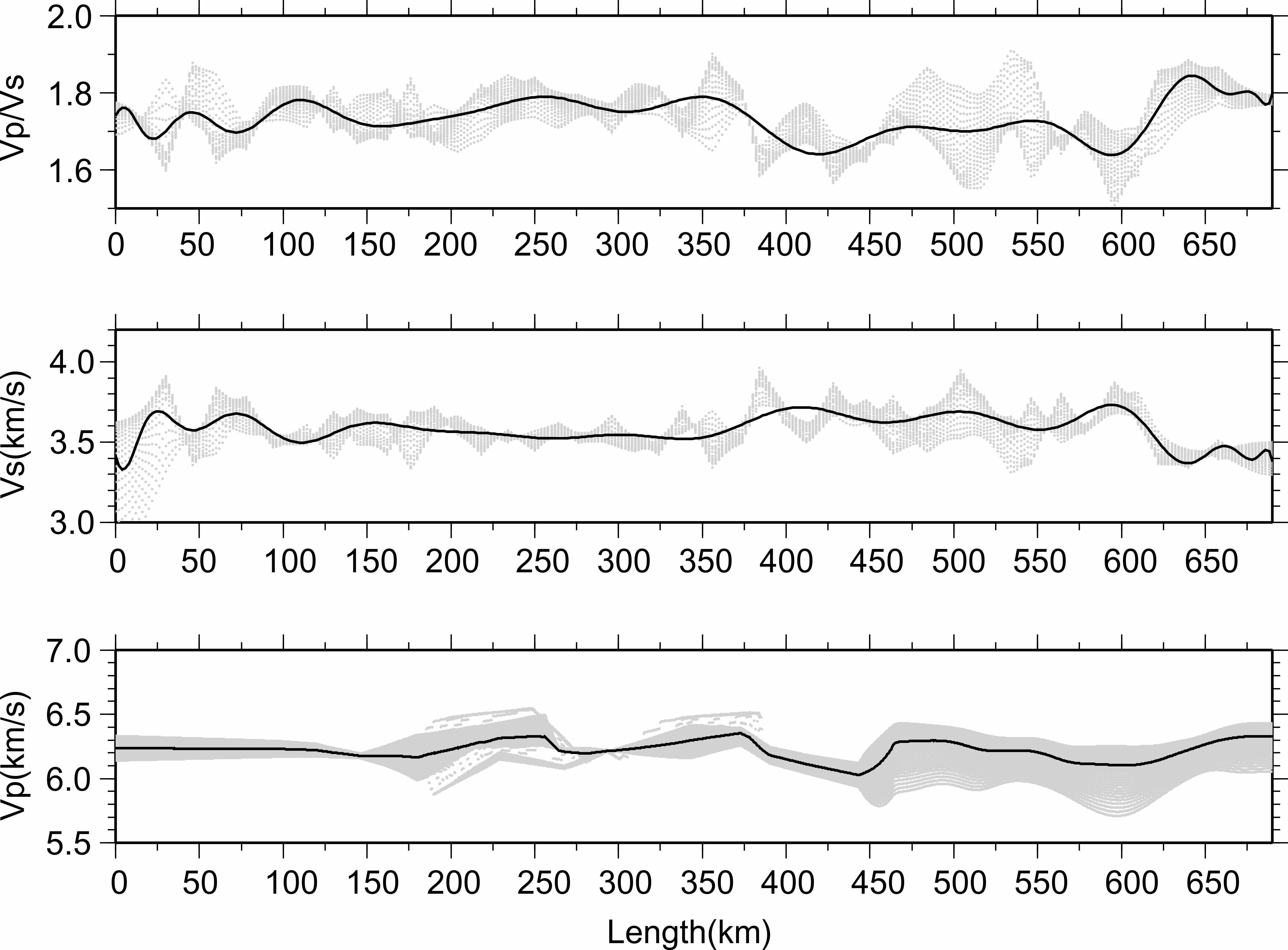
**

**Fig. S6.** Upper-crustal Vp, Vs, and Vp/Vs variations along profile AA’. Grey dots are data points extracted from our Vp, Vs, and Vp/Vs models (in Figs. 4 and S3c) within 8-18 km depths. Black curves are the results of arithmetic average (for Vp) or fitting a polynomial regression model using the least squares method (for Vs and Vp/Vs).


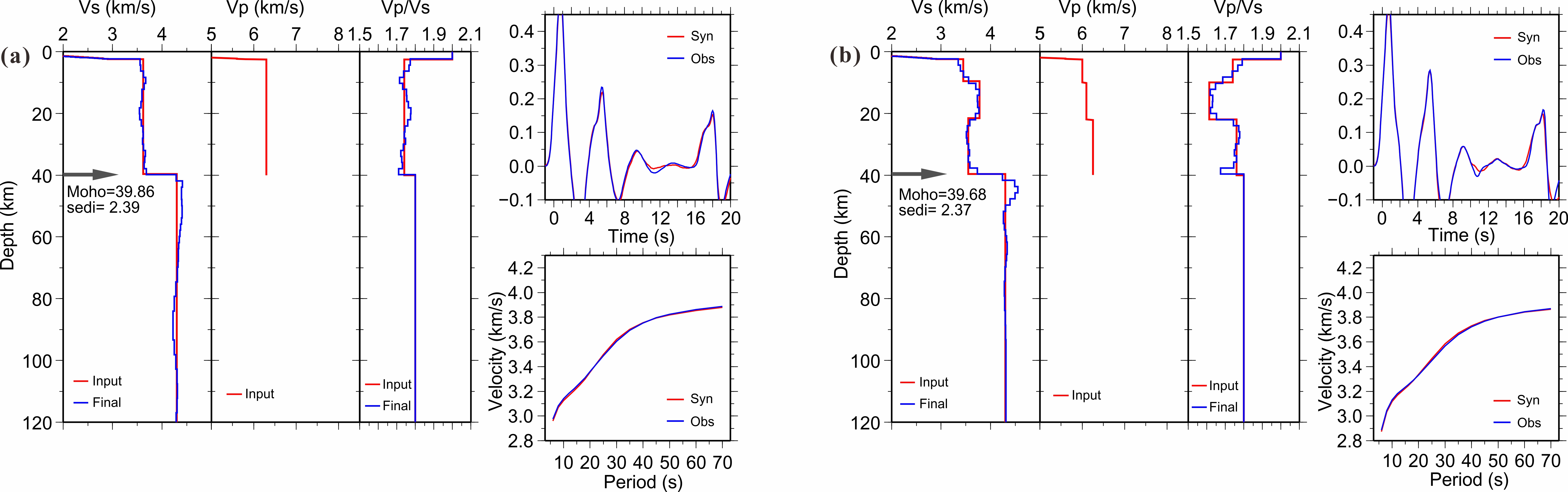


**Fig. S7.** Synthetic tests of the joint inversion regarding the effects of sediments. (a) The input model has a 40 km thick homogeneous crust with a 2.5 km thick sedimentary layer. (b) The input model has an 11 km thick upper-crustal high-velocity layer and a 2.5 km thick sedimentary layer. More synthetic cases regarding the effects of sediments were provided in Li *et al.* [4].


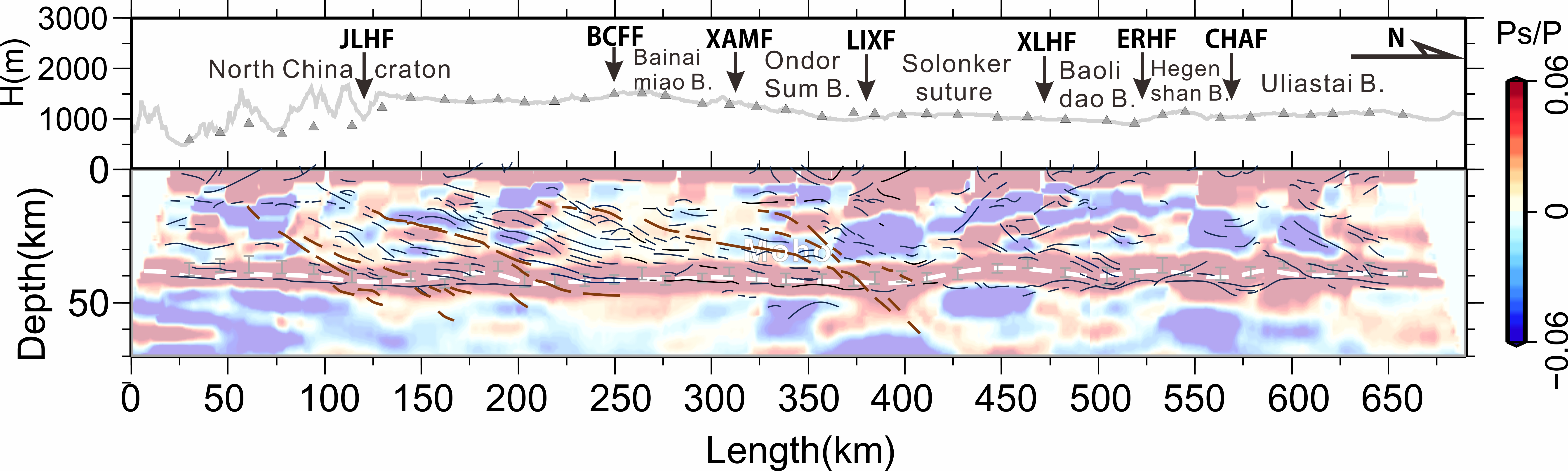


**Fig. S8.** Superimposition of the line drawings of high-amplitude reflectors (colored and black dashed lines) of the collinear deep-seismic-reflection profile (see Fig. 4d) on the broadband-seismic receiver-function profile.

**References**

1. Li WH, Keller GR and Gao R *et al.* Crustal structure of the northern margin of the North China Craton and adjacent region from SinoProbe02 North China seismic WAR/R experiment. *Tectonophysics* 2013; **606**: 116–26.

2. Li YK, Gao R and Yao YT *et al.* Crustal Velocity Structure from the Northern Margin of the North China Craton to the Southern Margin of the Siberian Plate (in Chinese with English abstract). *Chin J Geophys* 2014; **57**: 484–97.

3. Zhu LP and Kanamori H. Moho depth variation in southern California from teleseismic receiver functions. *J Geophys Res: Solid Earth* 2000; **105**: 2969-80.

4. Li JT, Song XD and Zhu LP *et al.* Joint Inversion of Surface Wave Dispersions and Receiver Functions with P Velocity Constraints: Application to Southeastern Tibet. *J Geophys Res: Solid Earth* 2017; **122**: 7291–310.
